# Diagnosing PrEP Communication Self-Efficacy in a Community-Based Peer Leader Intervention for Black Sexual Minority Men

**DOI:** 10.1007/s10461-022-03704-7

**Published:** 2022-05-18

**Authors:** Lindsay E. Young, Ashley Baird, John A. Schneider

**Affiliations:** 1 Annenberg School for Communication and Journalism, University of Southern California, Los Angeles, CA, United States; 2 Chicago Center for HIV Elimination, University of Chicago, Chicago, IL, United States; 3 Departments of Medicine and Public Health Sciences, University of Chicago, Chicago, IL, United States; 4 Crown School of Social Work Practice and Policy, University of Chicago, Chicago, IL, United States

**Keywords:** HIV prevention, Pre-exposure prophylaxis, Communication self-efficacy, Peer leaders, Social network interventions, Black/African american, Sexual minority men

## Abstract

HIV prevention interventions that leverage endogenous peer leaders to communicate about Pre-Exposure Prophylaxis (PrEP) and other HIV prevention tools in their social networks offer a way to reach greater portions of communities most impacted by HIV like Black/African American gay, bisexual, same gender-loving, and other sexual minority men (BSMM). However, the success of these interventions hinge on the communication self-efficacy of its peer leaders. In this exploratory study, we present a multi-theoretical framework that situates the PrEP communication self-efficacy (PCSE) of a cohort of young BSMM peer leaders (n = 303) in the context of personal, behavioral, social, and structural factors. Using censored regression models, our analysis shows that PCSE is influenced by evaluations of PrEP itself (its relative advantage, complexity, and compatibility), network embeddedness (degree centrality) among other BSMM, social media network exposure to HIV information, and medical mistrust. We conclude with a discussion of the practical implications of our findings for intervention design and implementation.

## Introduction

Black/African American gay, bisexual, same gender-loving, and other sexual minority men (hereafter BSMM) are more affected by HIV than any other group in the United States, accounting for 26% of the 37,968 new HIV diagnoses in 2018 [1]. Although HIV diagnoses have been stable overall among BSMM since 2014, diagnoses among young BSMM aged 25–34 increased by 12% between 2014 and 2018 [2]. As such, increasing BSMM’s access to and use of Pre-Exposure Prophylaxis (PrEP) — a medication to prevent HIV acquisition among HIV-negative individuals — has been made a key pillar of the United States’ *Ending the HIV Epidemic* plan [3].

However, despite its demonstrated efficacy in preventing HIV, PrEP use among BSMM has been limited. For example, in PrEP demonstration and implementation projects in Washington, DC [4] and New York City, NY [5], less than 15% of PrEP clients identified as Black. Additionally, only 31% of clients identified as Black in the CDC’s Sustainable Health Center Implementation PrEP Pilot (SHIPP) [6]. To put this into context, a recent study drawing on agent-based network models parameterized with data for young BSMM demonstrated that to meet ‘Getting to Zero’ timelines PrEP initiation among young BSMM, particularly those who are in serodiscordant relationships, would have to be closer to 40% [7].

An array of factors have been identified as barriers to PrEP use for BSMM, including common issues like concerns about PrEP side effects, uncertainty about PrEP’s effectiveness, and perceptions of personal HIV risk [8–10], as well as more upstream factors that disproportionately impact BSMM like socioeconomic vulnerabilities (e.g., unemployment), intersectional-, HIV-, and PrEP-related stigmas, and race-based medical mistrust [8, 11, 12]. In light of these realities, innovative engagement strategies are clearly needed that are capable of engaging greater portions of BSMM while addressing and/or circumventing noted barriers to prevention services.

Peer leader health interventions — where members of a prioritized population are positioned as health educators to promote the use of a health innovation in their networks [13] — offer opportunities to reach larger portions of BSMM at risk for HIV seroconversion. Further, they do so while privileging community-based systems of communication and influence over institutionalized systems that can engender mistrust. Indeed, efforts to leverage peer leaders to promote PrEP awareness and linkage among BSMM, although few in numbers, have shown promise in this regard [14–16].

Despite their potential, peer leader interventions have their challenges, particularly when the directive to peer leaders is to communicate about non-normative and potentially stigmatizing behaviors like PrEP [17]. The inherent difficulty in asking someone to initiate conversations with peers about a behavior that is unfamiliar or even controversial underscores a need to better understand sources of self-efficacy among these communicators and the degree to which their training in this role improves that self-efficacy.

Self-efficacy, or the beliefs one has about their capabilities to perform a task or execute a behavior [18], has served as an essential ingredient in many theories of health behavior change. Consequently, the concept of self-efficacy has received attention in HIV research as it relates to initiation and adherence to HIV medications like PrEP and anti-retroviral treatment [19–22]. Given the well-established relationship between self-efficacy and health behavior engagement, we argue that understanding sources of PrEP communication self-efficacy (hereafter PCSE) demands further attention, particularly from interventionists who aim to activate peer influence processes toward achieving greater PrEP engagement in high-incidence populations like BSMM. Further, Bandura himself argues that self-efficacy is not a dispositional determinant of behavior, but rather a contingent cognition affected by the situational interplay between beliefs, behaviors, and environmental influences [18]. Yet, despite this, research has rarely explored potential sources of self-efficacy at domains beyond personal factors [23].

**Fig. 1:**
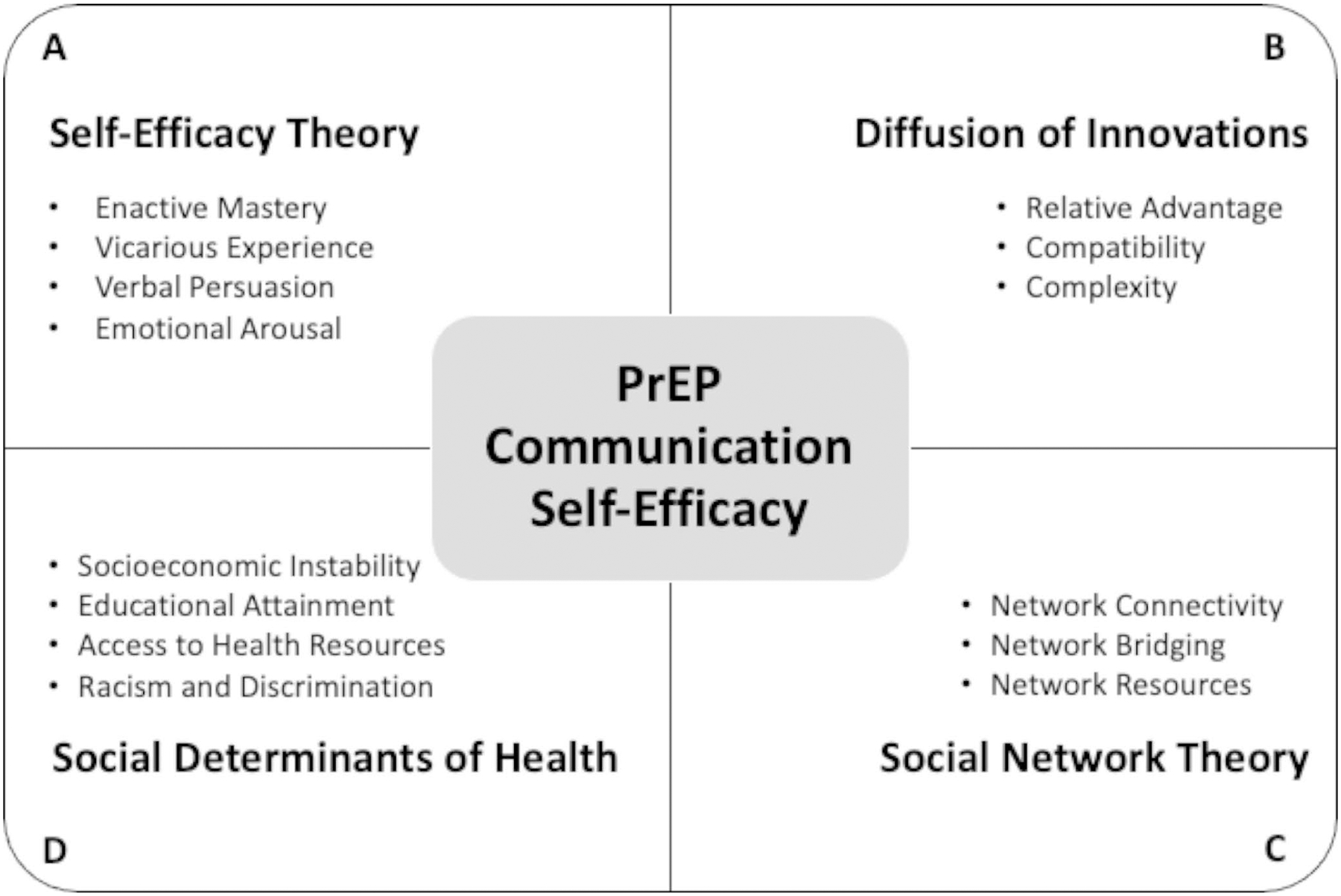
A multi-theoretical conceptual model of PrEP communication self-efficacy among young Black sexual minority men, including constructs associated with (clockwise from top left to bottom left): (A) Self-Efficacy Theory, (B) Diffusion of Innovations, (C) Social Network Theory, and (D) Social Determinants of Health

Using Self-Efficacy Theory as our starting point, we draw inspiration from three additional theoretical perspectives — diffusion theory, social network theory, and a social determinants of health framework — to expand our purview of potential domains and contexts that might influence BSMM’s confidence in their ability to discuss PrEP with peers (see Fig. 1). We then posit a series of factors to evaluate in relation to PCSE using data collected (2016–2018) from a large cohort of BSMM enrolled in a PrEP peer leadership intervention. Although exploratory in nature, the multi-theoretical lens that we adopt not only has implications for how self-efficacy is theorized but also for how peer leader interventions are designed.

## A Multi-Theoretical Perspective of PrEP Communication Self-Efficacy (PCSE)

### Self-Efficacy Theory

In his formulation of Self-Efficacy Theory [18], Bandura posits four factors that influence self-efficacy beliefs: (1) enactive mastery, (2) vicarious experience, (3) verbal persuasion, and (4) emotional arousal. *Enactive mastery* is related to an individual’s performance accomplishments with respect to the behavior of interest, which shapes their behavioral confidence and their behavioral persistence. The more practice an individual has performing a behavior, and the more successful that practice is, the more inclined they are to continue with it. *Vicarious experience* pertains to the indirect experience one gets with a behavior by observing others perform it. Observing people successfully perform a behavior, particularly when those people are similar to the focal individual, can boost an individual’s confidence that they, too, can perform the behavior [24]. *Verbal persuasion* occurs when an individual receives encouragement, instruction, and support from others, which is thought to motivate an individual to attempt and succeed in various behaviors. Finally, *emotional arousal* speaks to the feelings of stress, anxiety, or the anticipation of failure that an individual experiences while performing the behavior, which can decrease an individual’s confidence and the likelihood that they will persist in their practice of the behavior.

Although small in number, studies of HIV medication adherence have shown that having access to people who support an individual’s use and adherence to HIV medications [25–27] (verbal persuasion) and generalized mental health states like depression and anxiety [22, 28] (emotional arousal) positively and negatively contribute to their medication self-efficacy, respectively. Whether and how any of these four factors influence the self-efficacy to discuss such medications with peers has, to this point, been unexplored.

### Diffusion of Innovations

Diffusion of Innovations theory [29] underscores the dependencies between personal evaluations of innovation traits and the likelihood that an innovation will be adopted. Three of these traits are: (1) relative advantage, (2) compatibility, and (3) complexity. *Relative advantage* is the perception that an innovation offers a clear advantage over the idea or behavior that it supersedes. *Compatibility* is the degree to which an innovation is consistent with the existing values, experiences, and needs of a potential adopter. And, *complexity* speaks to the degree to which an innovation is perceived as being difficult to understand and use. With respect to PrEP use decisions, the anticipation is that individuals will be more likely to adopt PrEP if they: (1) see a relative advantage to taking PrEP over other prevention modalities, (2) view taking PrEP as compatible with their current sexual health care needs, and (3) consider PrEP to be simple and straightforward to take. Indeed, confidence in the merit of these perceived attributes is such that recommendations have been made to address them directly in PrEP messaging and PrEP interventions [30]. In this study, we explore whether these same evaluations impact BSMM’s competencies to promote PrEP among peers.

### Social Network Theory

The social network perspective posits that relationships influence a person’s attitudes, beliefs, and behaviors above and beyond the influence of their individual attributes [31]. As such, whom a person is connected to, how they are positioned within their social networks, and their access to network resources create contexts that ought to be considered viable sources of self-efficacy. Extant research provides clues as to how networks might relate to a person’s PCSE. First, Self-Efficacy Theory itself suggests that social networks are vital sources of social support, and social support plays a crucial role in increasing a person’s self-efficacy [18]. One way to achieve support is through embeddedness in networks with similar others, which has been linked to greater self-efficacy in performing behaviors that are encouraged by group members [32] and greater willingness to communicate with peers about sensitive issues like HIV prevention [17, 33]. Second, diffusion studies show that network bridges — individuals who occupy boundary-spanning positions within networks — are optimal for facilitating the spread of information across subcommunities and are known to be more open to discussing non-normative ideas and behaviors like PrEP [34]. Finally, research has shown that networks also serve as conduits of health communication and health information sharing [35]. To these ends, social media-based networks are particularly salient, as they enable circulation of information sourced by both peers and media outlets. Prior research has established a link between media exposure to HIV information and discussion of HIV in social networks [36, 37]. Further, if the HIV information that one is exposed to is sampled locally from an individual’s peer group, there is a greater chance that the individual will see the points of view expressed as being globally normative [38], which may reduce barriers to initiating HIV related conversations with peers.

### Social Determinants of Health

Social determinants of health are the “conditions in the environments where people are born, live, learn, work, play, worship, and age” that have downstream effects on health and are responsible for a wide range of health disparities [39]. A variety of social determinants, including access to health resources, poverty, and educational attainment have been identified as upstream barriers to HIV health care for BSMM [40]. At the root of many of these first-order structural impediments is a more pernicious one — structural racism. According to the CDC, experiences with racism and discrimination can negatively impact knowledge of HIV status, HIV care, and other needed services [41]. One response to direct and vicarious (e.g., intergenerational or social network stories) experiences of racial marginalization is medical mistrust [42, 43]. Defined as the lack of trust among marginalized groups in medical providers, the information they supply, and the institutions they represent [44], medical mistrust is conceived as a structural-level social determinant of health and health care disparities [45] with known implications for HIV-related health care via its negative impact on medication adherence [46], HIV testing engagement [47], and willingness to initiate PrEP [44]. Further, among African American women, medical mistrust has even been linked to health care self-efficacy [48].

An unexplored question as of yet, is whether medical mistrust can also have downstream effects on one’s willingness or confidence to advocate for an HIV medication like PrEP, an act that would require an individual to promote engagement with the very same health care system that is at the root of their mistrust. Therefore, we aim to explore whether medical mistrust as well as other known social determinants of health like socioeconomic instability, educational attainment, and access to health care resources impacts BSMM’s confidence in promoting a biomedical innovation like PrEP.

## Methods

### Study Design and Population

Data for this study derive from *PrEP Chicago*, a two-arm pragmatic randomized trial designed to test the efficacy of a novel community-level PrEP peer leader intervention. The intervention aimed to leverage the influence of a large cohort of newly trained BSMM peer leaders to increase PrEP awareness and linkage in their personal networks [49]. Participants were eligible if they (1) were 18–35 years of age, (2) identified as Black/African American, (3) were assigned male sex at birth, (4) had sex with a man in the past 12 months, and, because the intervention drew on social media as a communication tool, (5) had an active Facebook profile. People living with HIV were not excluded from participation, as the intervention aimed to motivate participants to promote PrEP in their personal networks, not necessarily to adopt it themselves. Once deemed eligible, individuals were assigned randomly to one of two conditions: (1) a treatment arm, whereby participants received the peer leader training and engaged in monthly check-in calls with study staff, or (2) an attention control arm, whereby participants took part in a half-day risk assessment workshop with no staff engagement following.

### Study Procedures

Recruitment occurred between March 2016 – March 2017 using respondent-driven sampling [50], a variant of snow-ball sampling that draws on referrals, beginning with a set of initial “seeds” that met study eligibility. Because seeds should have large social networks (i.e., are popular) and have ties to a diverse array of people belonging to different subpopulations [50–52], we selected our seeds based on their central or boundary spanning positions (i.e., structural signatures of popularity and diversity, respectively) within a previously derived Facebook friendship network among members of the target population [53]. These same traits – having large networks and ties to different subgroups – are also thought to be important characteristics of effective peer leaders [54]. While popular (or central) network actors have the status and connections needed to influence their peers [55, 56], those who span the boundaries of unique subcommunities are crucial in getting innovations to spread across regions of the network [57, 58]. Once a seed was enrolled and completed their baseline assessment, they were instructed to recruit up to 6 peers (or “sprouts”) who also met study eligibility criteria. Following enrollment, sprouts were also instructed to recruit peers who met study eligibility criteria, and the process continued until the recruitment target was reached. Participants received a cash incentive for each peer whom they successfully referred into the study. In total, 423 BSMM were successfully recruited into the study as a result of the respondent-driven sampling approach. Of those, 347 were retained at the 12-month assessment.

Participants consented to three types of data collection at baseline and 12-months. A computer-assisted self-administered survey included modules on PrEP knowledge, attitudes, and behaviors, sexual health behaviors, mental health, social media use, dispositions toward the medical and health care systems, and demographics. Biomedical testing determined participants’ HIV and syphilis status. And, to measure the impact of participants’ social connections on intervention outcomes, a list of Facebook friends was collected from each participant using Facebook’s manual data download feature. Several measures were taken to ensure that these data were collected using ethical best practices. First, to make sure that participants understood the Facebook download process, participants were walked through a unique consent form for the Facebook download that explained which types of Facebook data would be downloaded and the security measures that would be taken to ensure data protection. Second, for the third party (non-participant) Facebook friends of participants, a waiver of consent was obtained from the IRB given minimal risk to these individuals. However, additional data protections to secure third party identities (e.g., hashing, numeric identifiers) were also implemented prior to data analysis. It is also important to note that the data download does not grant researchers access to a Facebook friend’s profile or any personal information about those friends; it only provided access to the username itself. Finally, when it made sense to do so, we excluded non-participant network members and their ties to participants from the analysis all together, as we did when computing the network metrics for the featured study. All study procedures were approved by the Institutional Review Boards at University of Chicago Biological Sciences Division and NORC at the University of Chicago.

### The Peer Leader Training

The goal of the peer leader training was to build participants’ knowledge about PrEP and develop their PrEP communication skills. The intervention consisted of a half-day training, followed by monthly check-in calls (or “boosters”) with intervention staff. The peer leader training was adapted from the HIV Prevention Trials Network peer education and mentoring program [59] and included four modules: (1) HIV facts and myths; (2) PrEP education; (3) conversational role plays; and (4) leveraging social media to spread awareness about PrEP. Modules 3 and 4, specifically, were designed to develop participants’ communication self-efficacy and effectiveness.

### Intervention Evaluation

As described in greater detail elsewhere [15], the outcome on which the parent intervention was evaluated was a consolidated surrogate outcome that represented early phase PrEP care initiation among community members not enrolled in the study. Specifically, the consolidated outcome was operationalized on the basis of non-participant community members who were also linked to study participants via Facebook friendship ties, who over the observation period were either referred to the city-wide PrEP warmline or who attended a first PrEP care appointment.

Two separate analyses were performed to evaluate the intervention’s impact on the consolidated outcome: (1) a timing analysis of PrEP referrals after intervention sessions; and (2) a primary comparison of intervention and control conditions. Results from the timing analysis showed that during the 55-week observation period, a PrEP referral or appointment was most likely to occur within 3 days of an intervention training session compared to control (OR = 0.07, p = .007, 95% CI [0.02–0.013]), while results from the direct comparison analysis showed that non-participant community members with warmline referrals or appointments were more likely to be connected to study participants who had previously completed their peer leader training than participants in the control arm (aOR = 1.50, p = .012, 95% CI [1.09–2.06]).

The exploratory analysis performed here is part of a secondary wave of analyses motivated by the desire to better understand theorized mechanisms of peer leader influence, such as communication self-efficacy, and the factors that influence those mechanisms including the intervention itself.

### Measures

#### Outcome Measure

The outcome of interest is PrEP communication self-efficacy (PCSE) measured at 12-months and was operationalized as a mean composite score (*M* = 82.85, *SD* = 23.69) of two items measured on a 0–100 confidence scale [60] (“*How confident are you that you could talk about PrEP with your friends?”* (*M* = 83.23, *SD* = 24.34) and “*How confident are you that you could talk about PrEP with your sexual partners?”* (*M* = 83.02, *SD* = 27.21)). Cronbach’s alpha for the two communication self-efficacy items was 0.75, showing an internal consistency within the recommended 0.70–0.90 range of acceptability [61].

#### Multi-Theoretical Predictors

*Self-Efficacy Theory* To represent enactive mastery, we include two variables — *personal PrEP use* and *prior PrEP conversations —* that represent having prior performance experience with PrEP itself and the act of communicating about PrEP, respectively. Personal PrEP use was operationalized as whether a participant had current or past PrEP use experience at the 12-month assessment (13%). Prior PrEP conversations was measured as a count variable (0 to 4) of the number of distinct sources (friend, sex partner, doctor, HIV counselor) from whom the participant had heard and learned about PrEP since their baseline visit (*M* = 0.89, *SD* = 0.96). Although not an indicator of initiating PrEP conversations, being engaged in a conversation about PrEP initiated by someone else is a conversational experience nonetheless that a participant can learn from. A participant’s vicarious experience with PrEP was operationalized as the proportion of an individual’s study participant Facebook friends who had personal experience taking PrEP (*M* = 0.20, *SD* = 0.16). Receiving verbal persuasion to engage in PrEP-related communication was operationalized with an indicator variable representing whether a participant received the peer leader training in Year 1 of the study (51%). Finally, to represent a participant’s emotional arousal, we include a measure of anxiety using the 7-item generalized anxiety disorder scale (*M* = 13.15, *SD* = 6.16) [62]. That this is not a specific measure of the anxiety one feels when communicating about PrEP is an acknowledged limitation.

*Diffusion of Innovations* PrEP’s relative advantage was operationalized as an interval variable representing a participant’s perception that condoms are more effective than PrEP (1 = “Strongly Disagree” to 5 = “Strongly Agree”) (*M* = 2.89, *SD* = 1.16). In the absence of a measure that captures an individual’s personal evaluation of PrEP’s compatibility, we instead use an indicator variable for living with HIV (48%) as a proxy for compatibility, as PrEP is only for HIV negative individuals. We used confirmatory HIV testing data administered at 12-months to determine HIV status. Finally, perceptions of PrEP’s complexity was operationalized on the basis of a participant’s agreement that “*Taking PrEP is simple and straightforward”* (1 = “Strongly Disagree” to 5 = “Strongly Agree”) (*M* = 3.80, *SD* = 1.15).

*Social Network Theory* Network factors include measures of network embeddedness (i.e., degree centrality), network bridging, and network exposure to HIV information. Measures for degree centrality and network bridging were calculated from the Facebook friendship network among study participants. Degree centrality [63] was operationalized as the number of Facebook friendship connections that an individual has with other BSMM study participants (*M* = 31.11, *SD* = 25.52), and network bridging was operationalized using Everett & Valente’s calculation for network brokerage (*M* = 28.38, *SD* = 30.37) [64]. Network exposure to HIV information was operationalized as a numeric count (0 to 6) of the number of distinct social media platforms (Facebook, Twitter, Instagram, Snapchat, LinkedIn, Grindr) on which a participant was active and where they had come across HIV-related information or had been given advice by an online network associate about HIV prevention (*M* = 1.04, *SD* = 1.00).

*Social Determinants of Health* Educational attainment, employment status, access to health insurance, and a measure of medical mistrust are included as potential social determinants of PrEP communication self-efficacy. Educational attainment is defined categorically (1 = “less than high school” (9%), 2 = “high school diploma” (66%), 3 = “vocational certificate or Associate’s degree” (20%), 4 = “Bachelor’s degree or more” (5%)), employment status is represented as a binary measure of being unemployed (45%), and health coverage is defined as having health insurance (53%). Medical mistrust was measured using the 6-item group-based medical mistrust scale [65]. Scale items were measured on a five-point agreement scale and captured multiple aspects of psychometric-based medical mistrust experienced within the African American community, including perceptions of mistreatment based on race, perceptions of the (un)trustworthiness of medical information and providers, and perceptions of equitable treatment. Cronbach’s alpha for the six medical mistrust items was 0.71, which is within, but on the lower end, of the 0.70–0.90 range of acceptability [61]. An inspection of the item-rest correlations [66] for each of the six items revealed that the two reverse-coded items in the original six-item scale (“*Doctors have the best interests of Black people in mind* and *“Black people are treated the same as people of other groups by doctors or healthcare workers*”) were tenuously correlated with the remaining items. However, rather than drop these two items from the original scale, we opted to keep the scale intact as the alpha based on all six items was still within an acceptable range. A mean composite score of medical mistrust was calculated from the scores on the six items (*M* = 2.51, *SD* = 0.72).

#### Control Measures

All models were adjusted for a lagged measure of PCSE measured at baseline (*M* = 84.95, *SD* = 22.42) to account for potential temporal correlations. Summary statistics for all measures are shown in Table 1.

### Statistical Analysis

The dependent variable and all multi-theoretical factors were measured using 12-month data. The decision to use 12-month data as opposed to baseline data was motivated by two considerations. First, with 12-month data we could evaluate how the intervention itself (i.e., being trained as a PrEP communicator) impacted the PCSE of study participants, which, as described above, was not a planned component of the primary evaluation scheme of the intervention. We do this by including an indicator variable for being assigned to the treatment arm of the intervention as a proxy measure for Bandura’s concept of verbal persuasion. Our second motivation for using 12-month data was more practical. Medical mistrust, access to HIV information via social media, and generalized anxiety were only measured in the 12- and 24-month assessments. As such, we wanted to avoid the temporal complications of drawing from data collected at different time points during a participant’s enrollment.

In total, 347 of the 423 enrolled study participants were retained for the 12-month assessment, and only those who had complete data on all variables of interest (n = 303) were included in our analysis. Filtered cases (*n* = 44) did not differ significantly from the analytic sample (*n* = 303) by any of the key variables with the exception of exposure to HIV information on social media platforms. Individuals in the analytic sample tended to report more exposure to HIV information on social media platforms (*M* = 1.05, *SD* = 1.02) than individuals who had incomplete data (*M* = 0.77, *SD* = 0.61), *t*(344) = 1.75, p = .043.

Descriptive statistics and multivariate censored regression (Tobit) models were estimated using Stata version 17 [67]. Tobit models are appropriate when estimating linear relationships between variables when there is censoring from above or below in a continuous dependent variable [68]. Censoring from above occurs when cases with a value at or above some upper bound threshold take on the value of that threshold, so that the true value might be equal to the threshold, or it might actually be higher. Likewise, censoring from below occurs when values at or below some lower bound threshold are censored. In the case of PCSE, which we measure on a scale from 0 to100, censoring from above was observed as evidenced by the disproportionate number of cases, relative to the rest of the distribution, with self-efficacy scores equal to the upper bound of 100 (*n* = 145).

## Results

We estimate and compare four nested censored regression (Tobit) models, featuring relevant factors associated with the four theoretical perspectives that we posit have implications for understanding PrEP communication self-efficacy (PCSE). Modeling results are shown in Table 2. Model 1 is the baseline model and includes measures of the four factors underscored in Bandura’s original conceptualization of self-efficacy (i.e., enacted mastery, vicarious experience, verbal persuasion, and emotional arousal). In model 2 we add three factors associated with innovation adoption (i.e., the relative advantage, compatibility, and complexity of the innovation) that, by extension, could affect an individual’s confidence in advocating for the innovation. In model 3, we add three features of a person’s social embeddedness (i.e., their connectedness, their boundary spanning, and their exposure to network resources). And, in model 4, we add four social determinants of health factors known to have downstream effects on HIV prevention and care engagement.

All models are adjusted for a lagged measure of PCSE measured at baseline to control for potential temporal correlations. Further, all numeric measures (interval and continuous) were standardized to enable ease of interpretation. Also reported in Table 2 are omnibus goodness of fit statistics, including the Likelihood ratio (LR) chi2 and McKelvey & Zavoina’s Pseudo-R^2^, which is a recommended measure of explained variance for Tobit models [69].

### Model 1: SET Factors Only

Results of model 1 show that PrEP experience positively and significantly predicted PCSE (*β* = 21.51, p = .007, 95% CI [5.93, 37.08]). A participant’s aggregate prior experience talking about PrEP with friends, sex partners, doctors, and/or HIV counselors was also positively associated with PCSE, but only with marginal significance (*β* = 4.18, p = .091, 95% CI [−0.67, 9.03]). Not significant were a participant’s vicarious experience with PrEP via their study participant Facebook friends (*β* = −0.85, p = .714, 95% CI [−5.43, 3.72]), the training and encouragement they received to engage in PrEP conversations as measured by whether they received the peer leader training (*β* = 2.64, p = .590, 95% CI [−7.00, 12.30]), and their general anxiety (*β* = −1.112, p = .647, 95% CI [−0.46, 0.65]).

### Model 2: SET and DOI Factors

Of the three DOI factors included in this model, the perception that condoms were more effective than PrEP (i.e., relative advantage) was a positive and significant predictor in this model (*β* = −5.09, p = .038, 95% CI [−9.88, −0.29]), as was the perceived complexity of taking PrEP as measured by the perception that PrEP is simple and straightforward to use (*β* = 8.24, p = .001, 95% CI [3.55, 12.94]). Not significant was the compatibility (or lack thereof) of PrEP for peer leaders living with HIV (*β* = −7.43, p = .125, 95% CI [−16.95, 2.08]). Finally, with the DOI factors accounted for, PrEP experience remained significant (*β* = 18.28, p = .021, 95% CI [2.73, 33.83]).

### Model 3: SET, DOI, and SNT Factors

Of the two measures of network position, being connected to more young BSMM (network centrality) positively and significantly predicted PCSE (*β* = 7.19, p = .008, 95% CI [1.90, 12.48]), while being a network bridge did not (*β* = −0.76, p = .757, 95% CI [−5.60, 4.07]). Study participants’ access to HIV related information in their online networks was also a positive and significant predictor of PCSE (*β* = 5.45, p = .035, 95% CI [0.39, 10.50]). Finally, with social network factors accounted for, personal PrEP experience loses its significance (*β* = 13.50, p = .086, 95% CI [−1.94, 28.93]). Further, with the addition of social network factors, HIV status, which we use as a proxy for the DOI concept of innovation compatibility, becomes a positive predictor of PCSE (*β* = −10.93, p = .024, 95% CI [20.43,−1.42]). Measures for the other DOI factors relative advantage (*β* = −6.18, p = .024, 95% CI [−10.97, −1.38]) and complexity (*β* = 7.99, p = .024, 95% CI [3.39, 12.60]) also maintained their significance.

### Model 4: SET, DOI, SNT, and SDH Factors

Finally, with the addition of upstream social determinants of health (SDH) factors, we learn that greater medical mistrust was positively and significantly associated with a reduction in PCSE (*β* = −6.11, p = .015, 95% CI [(−11.01, −1.20]). Not significant were educational attainment, unemployment, and insurance status. Together, the inclusion of these social determinants had little impact on the magnitude and significance of the other theoretical constructs relative to their estimation in model 3. The one exception is that receiving HIV-related information in their online networks lost its significance (*β* = 4.83, p = .061, 95% CI [−0.22, 9.87]).

### Sensitivity Analysis by HIV Serostatus

Given known differences in circumstances and experiences between people living with HIV and those who are not, we performed sensitivity analysis to determine whether and how these differences impacted our findings. First, we performed tests of difference (Chi-square tests or t-tests) between serostatus groups on all key independent variables. We found that serostatus groups differed on three characteristics featured in our analysis: (1) PrEP use (current or past), (2) Facebook degree (i.e., number of BSMM Facebook friends), and (3) medical mistrust. Specifically, individuals living with HIV at the 12 month assessment were less likely to have had current or past PrEP experience (*X*^2^ (1, *N* = 303) = 22.42, *p* < .001), were connected to more BSMM on Facebook (HIV negative: *M* = 28.19, *SD* = 24.40; HIV positive: *M* = 34.30, *SD* = 26.41, *t*(301) = −2.10, p = .018), and reported less medical mistrust (HIV negative: *M* = 2.58, *SD* = 0.68; HIV positive: *M* = 2.43, *SD* = 0.75, *t*(301) = 1.90, p = .029). To determine how these differences impacted our main findings, we stratified the sample by HIV serostatus and estimated the same censored regression model (excluding HIV serostatus as a predictor) on each subsample. Of the three characteristics for which differences were found between HIV negative individuals and individuals living with HIV, Facebook degree was only significant for individuals living with HIV (*β* = 11.42, *p* = .002, 95% CI [4.27, 18.57]). And, although individuals living with HIV had less medical mistrust than HIV negative individuals, the negative effect of medical mistrust on PCSE was significant only for individuals living with HIV (*β* = −13.20, *p* < .001, 95% CI [−20.44, −5.95]). This suggests that individuals living with HIV in our sample might be more sensitive to their medical mistrust, despite having lower medical mistrust on average than their HIV negative counterparts.

## Discussion

In this study, we investigated PrEP communication self-efficacy (PCSE) in a large cohort of young BSMM, half of whom had undergone specific training to be a PrEP peer leader in their community. Our objective was to develop a more contextualized and culturally tailored picture of the domains and factors that affect this underexplored outcome, while also accounting for the effect of the intervention itself. Drawing on four theoretical perspectives that situate health behaviors and their correlates in multi-level contexts, we identified a series of factors across personal, social network, and structural domains and used censored regression models to determine their cumulative impact on PCSE.

Our analysis reveals several noteworthy trends that have implications for theory-building and practice. In the most naïve model, in which only factors underscored in Self-Efficacy Theory were included, only one personal experience stood out as a significant predictor of PCSE — having personal experience (current or past) using PrEP. However, as soon as factors associated with diffusion theory, social network theory, and a social determinants of health framework were incorporated into the model, this effect waned. Notably not significant were the effects of having peers who were on PrEP (i.e., vicarious experience), receiving formal motivation to be a PrEP communicator (i.e., verbal persuasion), and an individual’s generalized state of anxiety (i.e., emotional arousal). With respect to the null effect of verbal persuasion, Bandura concedes that encouragement from others is a weaker source of self-efficacy than those arising from one’s own accomplishments [18]. Nonetheless, a principle component of the parent intervention study was the verbal persuasion and ongoing motivational support that intervention staff provided those who were assigned to the treatment arm. That the participants who received this motivational support were no more likely than control participants to have greater PCSE after their first year of enrollment lends some support to Bandura’s claim. It may also be the case that the half-day peer leader training workshop was not adequately powered in terms of its intensity and frequency to influence the communication self-efficacy of peer leaders. Although more resource intensive and less pragmatic, hosting more frequent intermittent workshops and group check-ins during the duration of the intervention may be required to influence a peer leader’s confidence in this role through verbal persuasion.

In model 2, where factors implicated in diffusion theory were included, it became apparent that characteristics of the innovation itself (i.e., PrEP) were strongly associated with an individual’s confidence in communicating about that innovation. First, the perception that condoms were more effective than PrEP (i.e., relative advantage) was a consistent negative predictor of PCSE. This is a reminder that, for some, condoms remain the gold standard prevention modality. That the level of one’s PCSE is sensitive to this perception suggests that efforts to motivate individuals to talk about PrEP with peers might improve if PrEP were framed as a complement to condoms as opposed to an alternative.

Second, the degree to which an individual viewed PrEP as being easy to use (i.e., its complexity) was a consistent positive predictor of their PCSE. As such, the inverse is also true: those who perceive greater barriers to taking PrEP were less confident in their ability to promote it among peers. Given that BSMM, like most people, tend to have homophilous (i.e., like with like) social networks [70], it is possible that study participants whose own life circumstances made PrEP seem less viable also had peers who faced those same barriers. For these individuals, speaking to peers about PrEP may seem futile given what they know about their peers’ personal circumstances. Therefore, it seems essential that peer leader training curricula acknowledge relevant personal, social, and structural barriers to PrEP and help peer leaders devise cogent strategies for circumnavigating those barriers so that they are more convinced of its viability and, in turn, are more comfortable in broaching the subject with peers. Further, as taking PrEP gets simpler and more convenient with options like event-driven PrEP and longer-lasting injectable PrEP, this enhanced ease will become an important message for peer leaders to emphasize in their outreach.

Finally, we also learned that individuals living with HIV, which we use as a proxy for the perception that PrEP is incompatible with some individuals’ personal circumstances, tended to have lower PCSE once social network factors were accounted for (see model 3). Despite the perspectives that people living with HIV can bring to the task of promoting PrEP among HIV negative peers, that they themselves are ineligible for PrEP may make them feel less equipped to have those conversations. Adapting the training of peer leaders to speak more directly to the ways in which people living with HIV can benefit from others’ use of PrEP could be an effective strategy to increase their stakes in seeing PrEP diffuse in their community and to raise their confidence in being a persuasive agent to those ends. Further, as our subgroup analysis revealed, the medical mistrust of people living with HIV may be an additional barrier that needs further examination and redress.

In model 3, with social network features added, our results highlighted two features of an individual’s social embeddedness that influenced PCSE. First, individuals who had more social connections with other BSMM in the study tended to report greater confidence in their ability to communicate about PrEP in their social circles. In many ways, this is unsurprising. Having more BSMM friends may be suggestive of having greater social support for communicating about HIV prevention and having greater access to a pool of people who are themselves good candidates for PrEP, which may make it easier to initiate PrEP conversations. Further, this result suggests that PCSE itself may be a mechanism through which a peer leader’s social network position impacts their leadership effectiveness. Network centrality vis-à-vis other members of a target community is a common criteria for selecting peer leaders, as it reflects an individual’s status within the network and, therefore, their influence potential [54]. And, indeed, as shown elsewhere by the authors [71], PrEP peer leaders with more pre-existing relationships with other BSMM were connected to more people who were ultimately linked to PrEP care. Findings here suggest that PCSE could be a potential mediator of that relationship and should be examined as such in future research. However, it is important to note that with the status that comes with being well-connected comes pressures to conform to social standards [72]. As such, it is unclear how this embeddedness would operate if the innovation had been even more stigmatizing or taboo.

More than just manifestations of relationships, social networks are also sources of tangible resources. To this point, our analysis showed that a second social source of PCSE was the HIV-related information and advice that participants received from members of their social media networks. As prior work has found, exposure to information shared in one’s local peer networks can increase the perception that the points of view expressed in that information are accepted globally [38]. This perception of normativity may explain why increased exposure to HIV information strengthened participants’ confidence in initiating PrEP conversations with peers. The implication of this finding for intervention design is clear: increasing peer leaders’ exposure to information about PrEP and HIV prevention may have a positive cumulative effect on their communication self-efficacy over the course of their tenure in the peer leader role. To these ends, interventions would benefit from formalizing informational support in its mentoring of peer leaders. For example, as was done in the parent intervention, a centralized social media group for peer leaders could be curated where novel PrEP and HIV-related content could be circulated and, in turn, shared with non-participant peers.

Finally, our results underscore the importance of considering broader structural circumstances that can condition BSMM’s health outcomes, beliefs and behaviors. Specifically, we learned that participants with greater medical mistrust — a known contributor to disparities in HIV treatment and prevention engagement in marginalized communities [12, 44, 47, 73, 74] — were significantly more likely to report feeling less confident in their ability to advocate for PrEP. The discordance between one’s personal beliefs about medical institutions and the biomedical nature of the innovation they are being asked to promote is almost certainly at the root of this finding. At its core, this finding surfaces a reality that has yet to be adequately addressed by health behavior interventions implemented in African American communities. Generally speaking, interventions that draw on community-based assets like peer health leaders are not designed to be treatments for deeply rooted mistrust in institutionalized medical care. Instead, they more realistically present a means to circumnavigate that mistrust. Bearing this in mind, our findings suggest that when left unacknowledged and unaddressed, medical mistrust can linger and create a barrier to intervention efforts through its effect on BSMM’s PrEP intentions and/or through their self-efficacy in promoting PrEP in their community.

For this reason, PrEP interventions for BSMM may need to expand their curricula to address the realities of medical mistrust. Most importantly, medical interventionists should engage in a meaningful dialogue with participants about the historical and contemporary roots of medical mistreatment, while also creating safe spaces where participants can express personal reservations about the medical community. Second, interventionists should work with participants to proactively devise strategies for engaging with medical providers that leave them feeling more empowered and in control of their health care. Although not a panacea for medical racism as a whole, these small but intentional efforts to hear, acknowledge, and counteract BSMM’s experiences with medical discrimination could have downstream positive effects on their willingness to advocate for PrEP and other prevention modalities in their communities.

What we learned from our analysis must also be interpreted in the context of our data limitations. First, given that communication self-efficacy was not the intended outcome of the parent study intervention, our measures for the outcome itself and several of the multi-theoretical factors that we modeled err on the side of being over-simplified. This is particularly true for our measures of three factors stemming from self-efficacy theory (i.e., enactive mastery, verbal persuasion, and emotional arousal) and the concept of innovation (i.e., PrEP) compatibility as articulated in diffusion theory. That said, the goal of this analysis was exploratory in nature with the hope that our findings could motivate future self-efficacy studies designed for a more thorough and formal evaluation of the multi-theoretical constructs we explore in this paper. Second, we draw on online network data collected from participants to operationalize their social embeddedness (i.e., their central and boundary-spanning network positions). Although Facebook friendships and physical world relationships have a tendency to overlap [75, 76], the fact that neither measure of network position used in this study accounted for other, perhaps more relevant, relationships (e.g., confidants or sex partners) should be noted. Third, we limited our analysis to capturing only the direct relationships between the multi-theoretical factors and PCSE, leaving potential indirect and moderated pathways unaddressed. Future research should be directed toward investigating these more complex pathways. Finally, self-efficacy is only one factor of many likely to influence peer leaders’ successful advocacy of PrEP within social networks. Peer leaders’ communication styles and skills, their frequency of interaction and type of relationship with a given peer, and many other characteristics certainly contribute to successful PrEP communication advocacy. Understanding how PrEP communication self-efficacy and the factors associated with it interact with other mechanisms of effective peer leadership is an area of research that could yield tremendous insights for future intervention design.

Despite these limitations, this study is the first to our knowledge to examine PrEP communication self-efficacy as an outcome of interest and to situate it in the personal, social, and structural circumstances of a large cohort of BSMM as well as within the context of the peer leadership intervention itself. Although self-efficacy operates as a cognitive mechanism of task/behavior performance, our analysis clearly shows that confidence to discuss PrEP with peers arises from larger social and structural environments in which BSMM are embedded. Notably, we find that BSMM’s perceptions of PrEP itself, their social embeddedness among other BSMM, and their trust in medical providers and institutions influence their beliefs that they can effectively communicate about PrEP with peers. Using organic social networks and peer influence processes to spread awareness and encourage adoption of PrEP offers the opportunity to reach larger portions of BSMM with potentially life-saving prevention tools. However, these efforts necessarily depend on members of this community feeling competent and confident in their ability to communicate about PrEP with their peers. As such, understanding how the circumstances of BSMM’s lives facilitate or impede their confidence in having those conversations can provide crucial insights for selecting and training peer leaders and other lay health educators, thereby increasing the efficacy of peer-driven intervention programs.

## Figures and Tables

**Table 1:** Characteristics of Black Sexual Minority Men (BSMM) (*n* = 303): Chicago, IL, USA

Characteristics of BSMM	Mean (SD)
*Self-Efficacy Factors*	
Personal current or past PrEP use (enactive mastery) (0,1)	0.13 (0.34)
Prior PrEP conversations (enactive mastery)	0.89 (0.96)
Friends’ PrEP experience (vicarious experience)	0.20 (0.16)
Peer leader training (verbal persuasion) (0,1)	0.51 (0.50)
Anxiety (emotional arousal)	13.15 (6.16)
*Diffusion of Innovations Factors*	
Condoms better than PrEP (relative advantage)	2.89 (1.16)
Living with HIV (compatibility) (0,1)	0.48 (0.50)
Ease of PrEP use (complexity)	3.80 (1.15)
*Social Network Factors*	
Degree centrality (connectedness)	31.11 (25.52)
Network bridging (boundary-spanning)	28.38 (30.37)
Network exposure to HIV information (network resources)	1.04 (1.00)
*Social Determinants of Health Factors*	
Educational Attainment	
Less than high school (0,1)	0.09 (0.28)
High school diploma or equivalent (0,1)	0.66 (0.47)
Vocational or Associate’s Degree (0,1)	0.20 (0.40)
Bachelor’s degree or more (0,1)	0.05 (0.22)
Unemployed (0,1)	0.45 (0.50)
Health Insurance (0,1)	0.53 (0.50)
Medical Mistrust	2.51 (0.72)
*Self-efficacy Outcome*	
PrEP Communication Self-efficacy (12-months)	82.85 (23.69)
Lagged PrEP Communication Self-efficacy (baseline)	84.95 (22.43)

**Table 2:** Multi-theoretical nested censored regression models of PrEP communication self-efficacy (PCSE) featuring components of Self-efficacy Theory (SET), Diffusion of Innovations Theory (DOI), Social Network Theory (SNT), and Social Determinants of Health (SDH)

	Model 1: SET only	Model 2: SET + DOI	Model 3: SET + DOI + SNT	Model 4: SET + DOI + SNT + SDH
Variable (represented construct)	Coef (95% CI)	Coef (95% CI)	Coef (95% CI)	Coef (95% CI)
*Self-Efficacy Theory*				
Personal (current or past) PrEP use (enacted mastery)	21.51 (5.93, 37.08)**	18.28 (2.73, 33.83)*	13.50 (−1.94, 28.93)†	14.58 (−0.88, 30.04)†
Prior PrEP conversations (enacted mastery)	4.18 (−0.67, 9.03)†	3.72 (−0.98, 8.42)	1.48 (−3.27, 6.23)	1.63 (−3.08, 6.34)
Friends’ PrEP experience (vicarious experience)	−0.85 (−5.43, 3.72)	−1.09 (−5.54, 3.37)	−2.44 (−6.83, 1.94)	−2.16 (−6.49, 2.18)
Peer leader training (verbal persuasion)	2.64 (−7.00, 12.30)	0.79 (−8.59, 10.16)	−2.30 (−11.66, 37, 7.05)	−3.33 (−12.73, 6.07)
Anxiety (emotional arousal)	−1.12 (−0.46, 0.65)	−0.73 (−5.39, 3.92)	−0.03 (−4.64, 4.58)	0.82 (−3.84, 5.48)
*Diffusion of Innovations*				
Condoms better than PrEP (relative advantage)		−5.09 (−9.88, −0.29)*	−6.18 (−10.97, −1.38)*	−5.38 (−10.23, −0.53)*
Living with HIV (compatibility)		−7.43 (−16.95, 2.08)	−10.93 (−20.43, −1.42)*	−12.05 (−21.63, −2.48)*
Ease of PrEP use (complexity)		8.24 (3.55, 12.94)**	7.99 (3.39, 12.60)**	6.65 (1.99, 11.30)**
*Social Network Theory*				
Degree centrality (connectedness)			7.19 (1.90, 12.48)**	7.32 (2.04, 12.60)**
Network bridging (boundary-spanning)			−0.76 (−5.60, 4.07)	−0.07 (−4.88, 4.74)
Network exposure to HIV info (network			5.45 (0.39, 10.50)*	4.83 (−0.22, 9.87)†
resources)				
*Social Determinants of Health*				
Educational Attainment (ref = high school)				
Less than high school				−5.74 (−21.84, 10.37)
Vocational Cert/Associate’s Degree				5.57 (−6.20, 17.34)
Bachelor’s Degree or more				−7.78 (−28.74, 13.18)
Unemployed				−5.21 (−14.64, 4.22)
Health Insurance				0.95 (−8.29, 10.19)
Medical mistrust (Structural racism)				−6.11 (−11.01, −1.20)*
Lagged Dependent Variable	12.30 (7.41, 17.20)***	11.08 (6.29, 15.87)***	10.02 (5.35, 14.70)***	8.88 (4.24, 13.53)***
Observations (*N*)	303	303	303	303
Log likelihood (smaller is better)	−818.35	−810.05	−802.44	−797.94
LR chi2 (df)	37.36(6)***	53.96(9)***	69.18(12)***	78.19(18)***
McKelvey & Zavoina’s Pseudo *R*^*2*^	0.14	0.21	0.27	0.30

† p < .10;

* p < .05;

** p < .01;

*** p < .001

## Data Availability

For information about data access, please contact the corresponding author.
